# Strong near band-edge excited second-harmonic generation from multilayer 2H Tin diselenide

**DOI:** 10.1038/s41598-021-94612-8

**Published:** 2021-07-22

**Authors:** Rabindra Biswas, Medha Dandu, Asish Prosad, Sarthak Das, Sruti Menon, Jayanta Deka, Kausik Majumdar, Varun Raghunathan

**Affiliations:** Department of Electrical Communication Engineering, Indian Institution of Science, Bangalore, 560012 India

**Keywords:** Applied optics, Optical materials and structures

## Abstract

We report strong second-harmonic generation (SHG) from 2H polytype of multilayer Tin diselenide (SnSe_2_) for fundamental excitation close to the indirect band-edge in the absence of excitonic resonances. Comparison of SHG and Raman spectra from exfoliated SnSe_2_ flakes of different polytypes shows strong (negligible) SHG and Raman E_g_ mode at 109 cm^−1^ (119 cm^−1^), consistent with 2H (1T) polytypes. The difference between the A_1g_–E_g_ Raman peak positions is found to exhibit significant thickness dependent for the 1T form, which is found to be absent for the 2H form. The observed thickness dependence of SHG with rapid oscillations in signal strength for small changes in flake thickness are in good agreement with a nonlinear wave propagation model considering nonlinear polarization with alternating sign from each monolayer. The nonlinear optical susceptibility extracted from SHG signal comparison with standard quartz samples for 1040 nm excitation is found to be more than 4-times higher than that at 1550 nm. This enhanced nonlinear response at 1040 nm is attributed to the enhanced nonlinear optical response for fundamental excitation close to the indirect band-edge. We also study SHG from heterostructures of monolayer MoS_2_/multilayer SnSe_2_ which allows us to unambiguously compare the nonlinear optical response of SnSe_2_ with MoS_2_. We find the SHG signal and any interference effect in the overlap region to be dominated by the SnSe_2_ layer for the excitation wavelengths considered. The comparison of SHG from SnSe_2_ and MoS_2_ underscores that the choice of the 2D material for a particular nonlinear optical application is contextual on the wavelength range of interest and its optical properties at those wavelengths. The present works further highlights the usefulness of near band-edge enhancement of nonlinear processes in emerging 2D materials towards realizing useful nanophotonic devices.

## Introduction

Two dimensional (2D) materials with their novel opto-electronic properties continue to garner a lot of interest in the field of nonlinear optics^1,2^. Monolayer and multilayer 2D materials exhibit strong nonlinear optical response owing to their highly ordered crystalline structure, interesting layer number, polarization dependence^3^, and offer the possibility of incorporating electrical tunability^4,5^. These properties have led to 2D materials being proposed for use as ultra-thin active photonic devices for wavelength conversion, saturable absorption, optical limiting, optical modulation, parametric down-conversion etc.^1^ Second-harmonic generation (SHG) is one such nonlinear wavelength-conversion process in which high intensity laser illumination at the fundamental excitation is up-converted to twice the input frequency. Many of the transition metal dichalcogenides (TMDCs) exhibit extraordinary SHG response due to their layer number dependent non-centrosymmetry^3,6,7^. The nonlinear optical response from TMDCs are strongly enhanced near their excitonic resonances^4,5^. The nonlinear optical response is also expected to be enhanced close to direct or indirect band-edges due to the fundamental excitation being close to real states with increased density-of-states near the band-edge^8,9^. Such band-edge enhancement of nonlinear optical processes has been reported previously in bulk semiconductors^10,11^, but not reported for 2D materials, too the best of our knowledge. In this paper we report strong SHG from 2H polytype multilayer Tin diselenide (SnSe_2_) for fundamental excitation in the vicinity of the indirect band-edge.

SnSe_2_ is a group IV-VI metal dichalcogenide with a hexagonal lattice structure^12^. Layered SnSe_2_ is known to exist in both 1T and 2H polytype forms with D_3d_ and D_3h_ symmetry respectively^13,14^. The crystals with D_3d_ and D_3h_ symmetry exhibits layer number independent centrosymmetry, and odd-layer non-centrosymmetry respectively. The indirect and direct energy bandgaps of SnSe_2_ are in the range of 1.07–1.59 eV and 1.84–2.04 eV respectively, depending on the layer number^9^. Multi-layer SnSe_2_ with layer number greater than ten exhibits indirect bandgap close to the bulk form (~ 1.07 eV)^8^. There are no excitonic resonance features observed in either monolayer or multilayer form, leading to band-to-band transitions being the dominant optical process. In the context of nonlinear optical studies, saturable absorption using multilayer SnSe_2_ for nanosecond and sub-picosecond optical pulse generation has been reported previously^15–17^. We have previously reported multi-photon photoluminescence enhancement in monolayer-MoS_2_/multilayer-SnSe_2_ heterostructures due to a resonant energy transfer mechanism between the strongly absorbing multilayer SnSe_2_ and the light emitting monolayer MoS_2_^18^. We have also studied thickness dependent forward and backward generated third-harmonic generation from multilayer SnSe_2_^19^.

For studying the nonlinear optical properties of 2D materials, nonlinear optical microscopy has been a particularly useful tool. This has been applied to identify crystal structure^20^, grain boundaries^21^, sample inhomogeneity^22^, and twist-angle between artificial stacked layers^23^. In the present work, we make use of SHG microscopy to acquire spatio-spectral maps of multilayer SnSe_2_ flakes and its heterostructures to determine the thickness, polarization, and excitation wavelength dependence of the SHG signal. First, we compare the SHG signal strength and Raman spectra from samples of different polytypes of SnSe_2_ and observe strong (negligible) SHG combined with E_g_ Raman mode observed at 109 cm^−1^ (119 cm^−1^). These observations correlate well with the properties of 2H and 1T polytypes of SnSe_2_. The difference between the A_1g_–E_g_ Raman peak positions is found to exhibit strong thickness dependent for the 1T form, which is absent for the 2H form. We observe rapid oscillations in SHG signal with small changes in flake thickness combined with a gradual decrease in the SHG signal with increased thickness. The thickness dependent SHG agrees well with a nonlinear-wave propagation model considering layered medium with alternating signs for the nonlinear optical susceptibility of the individual monolayers. Comparative SHG measurements with quartz is used to extract the nonlinear optical susceptibility. The second-order nonlinear optical susceptibility at 1040 nm (1.19 eV) excitation is found to be more than 4-times higher than that at 1550 nm (0.8 eV) excitation for SnSe_2_, which has an indirect band gap in the range of 1.07–1.59 eV. This is attributed to the enhancement of SHG for fundamental excitation in close vicinity of the indirect band-edge. SHG images of heterostructures of monolayer MoS_2_/ multilayer SnSe_2_ are used to compare the SHG signal strengths and understand SHG interference in the overlap region. The SHG signal from the multilayer SnSe_2_ considered here is ~ 34 times stronger than monolayer MoS_2_ for 1040 nm excitation. This is observed despite considering monolayer MoS_2_ having a direct bandgap at 1.9 eV^18^, which is expected to give the strongest SHG signal for layered MoS_2_. The present work underscores the usefulness of near band-edge enhancement of nonlinear optical processes in emerging 2D materials, such as SnSe_2_ with potential applications in realizing ultra-thin nonlinear optical devices.

## Results and discussion

### Comparison between two common polytypes of SnSe_2_

**Figure 1:**
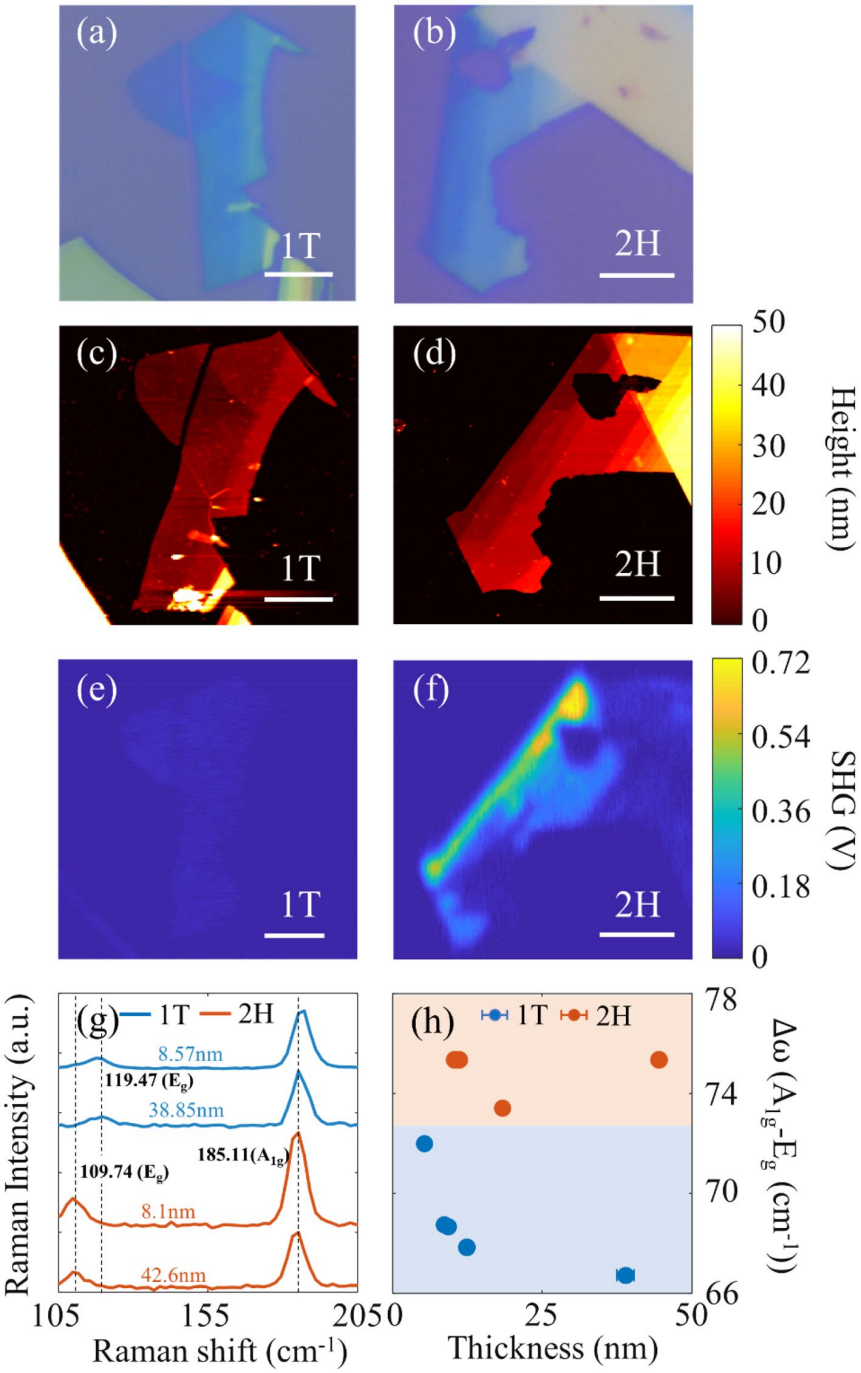
Optical images of: (**a**) 1T, (**b**) 2H polytype of SnSe_2_. AFM images of: (**c**) 1T, (**d**) 2H polytypes of SnSe_2_. SHG images of: (**e**) 1T, (**f**) 2H polytypes of SnSe_2_. Scale bar for all images is 5 μm (**g**) Raman spectra of 1T (blue solid curves) and 2H (orange solid curves) sample, the dashed black line indicates the Raman peak positions of the E_g_ and A_1g_ Raman modes. (**h**) Thickness dependent A_1g_–E_g_ Raman peak separation for SnSe_2_, 1T (blue solid circles) and 2H (orange solid circles). (Created in MATLAB v9.6, R2019a, https://in.mathworks.com/).

First, we perform SHG measurements and Raman spectroscopy on SnSe_2_ flakes (12 samples in total) from two different sources, as described in the “Methods” section below. We observe clear clustering of the samples into two groups based on the SHG signal strength, which is found to correlate well with the observed Raman peak positions and its thickness dependence. This clustering is found to be consistent with the 1T and 2H polytypes of SnSe_2_. Figure 1 shows a representative comparison of the optical, atomic force microscopy (AFM), SHG images and Raman spectroscopy measurement acquired for multilayer SnSe_2_ with the two distinct polytypes. The optical (Fig. 1a, b) and AFM (Fig. 1c, d) images of the two samples show very similar color contrast and thickness profiles. The SHG images (Fig. 1e, f) however show signal strengths which are different by almost an order of magnitude. The observed difference in SHG signal strength between the two samples is consistent with the centrosymmetric 1T polytype exhibiting negligible SHG in comparison to the non-centrosymmetric 2H polytype exhibiting strong SHG for odd number of layers. This observation is further corroborated by Raman spectroscopy measurements. Raman scattering is known to be sensitive to material phase and has been used for identifying different polytypes in TMDCs^24,25^. Raman spectra acquired for both thin and thick regions of the two samples are shown in Fig. 1g. The in-plane (E_g_) Raman mode for the two samples are located at ~ 109 cm^−1^ and ~ 119 cm^−1^ respectively. This is consistent with previous reports of Raman spectra from 2H and 1T polytypes of SnSe_2_^14,26^. The out-of-plane (A_1g_) Raman mode is obtained at ~ 186 cm^−1^ for both polytypes with slight blueshift with increasing thickness. The thickness dependence of the Raman peak separation between the A_1g_ and E_g_ modes, shown in Fig. 1h also exhibits clear difference between the two samples. The error bar in the AFM thickness measurement of each data point considers the variation of thickness over an area of 0.4 × 0.4 µm^2^ with a pixel resolution of 80 nm. For the 1T polytype, the A_1g_–E_g_ peak separation is found to decrease with increasing layer thickness (blue shaded area in Fig. 1h). This is consistent with the previous report of Raman spectroscopy on SnSe_2_ nanoflakes^14^. The 2H polytype however shows negligible thickness dependence of A_1g_–E_g_ separation (orange shaded area in Fig. 1h). This observation for the 2H polytype has not been reported previously, to the best of our knowledge.

Recent reports have also shown that SnSe_2_ can transform to Tin monoselenide (SnSe) due to thermal annealing^27^. SnSe is known to also exhibit strong SHG^28^. The observed Raman peak positions obtained from the sample after the SHG studies are consistent with that of SnSe_2_ and shows that the sample does not undergo stoichiometric conversion to SnSe. In the rest of the experimental study presented in this paper, we focus only on 2H SnSe_2_ due to the strong SHG signals obtained for this polytype.

### Thickness dependent SHG from 2H SnSe_2_

**Figure 2:**
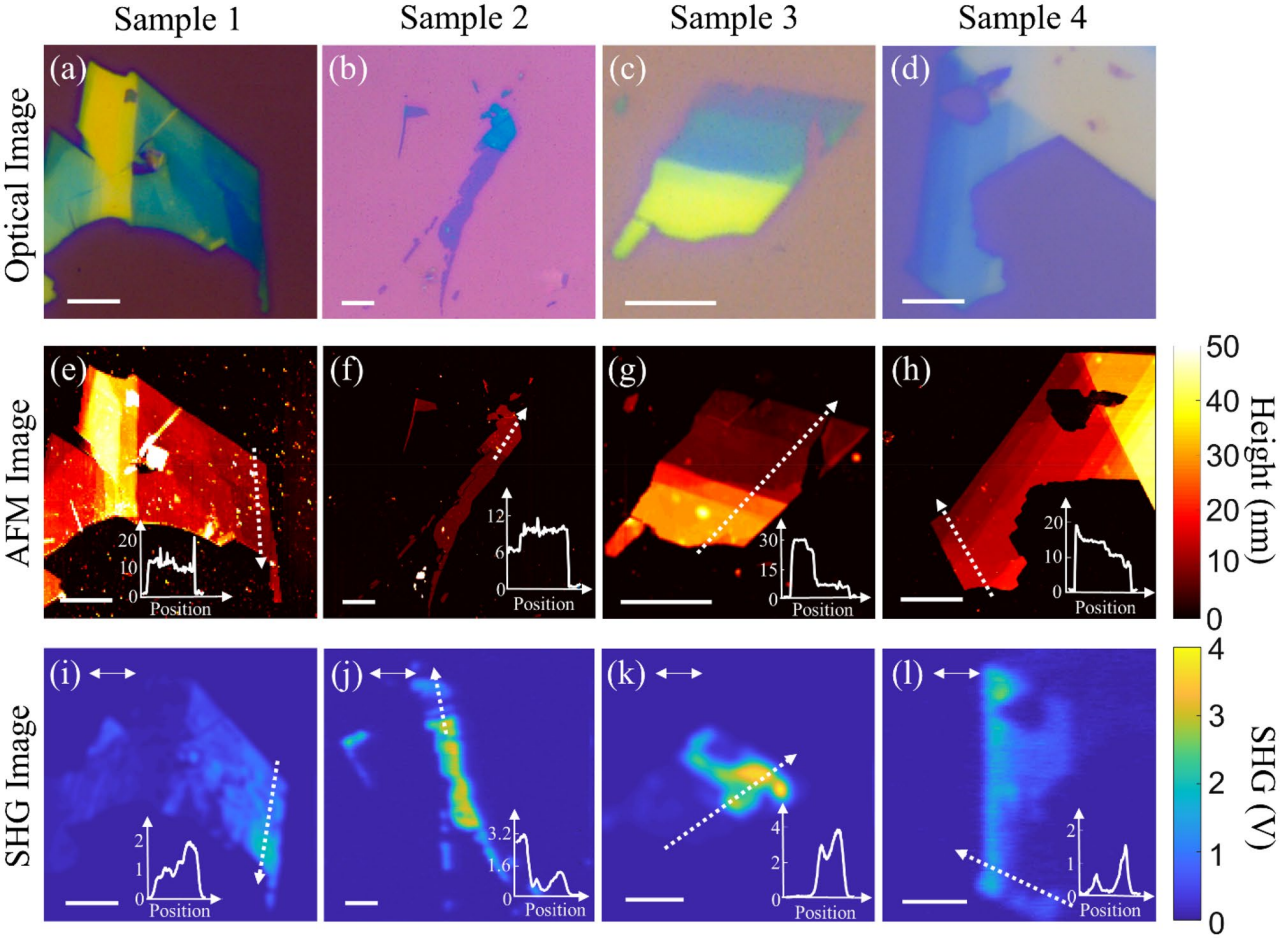
Summary of images acquired for four different 2H SnSe2 samples using different imaging modalities. (**a**–**d**) optical reflectance images, (**e**–**h**) AFM image and (**i**–**l**) SHG image with incident light polarized along the arm-chair axis. Each line-scan shown in the inset of AFM and SHG image are along the white dashed line marked in the images with arrow points the direction of the line-scan. Scale bar shown in all images is 5 μm. The double-headed arrows shown in the SHG Images correspond to the incident polarization direction. (Created in MATLAB v9.6, R2019a, https://in.mathworks.com/).

**Figure 3:**
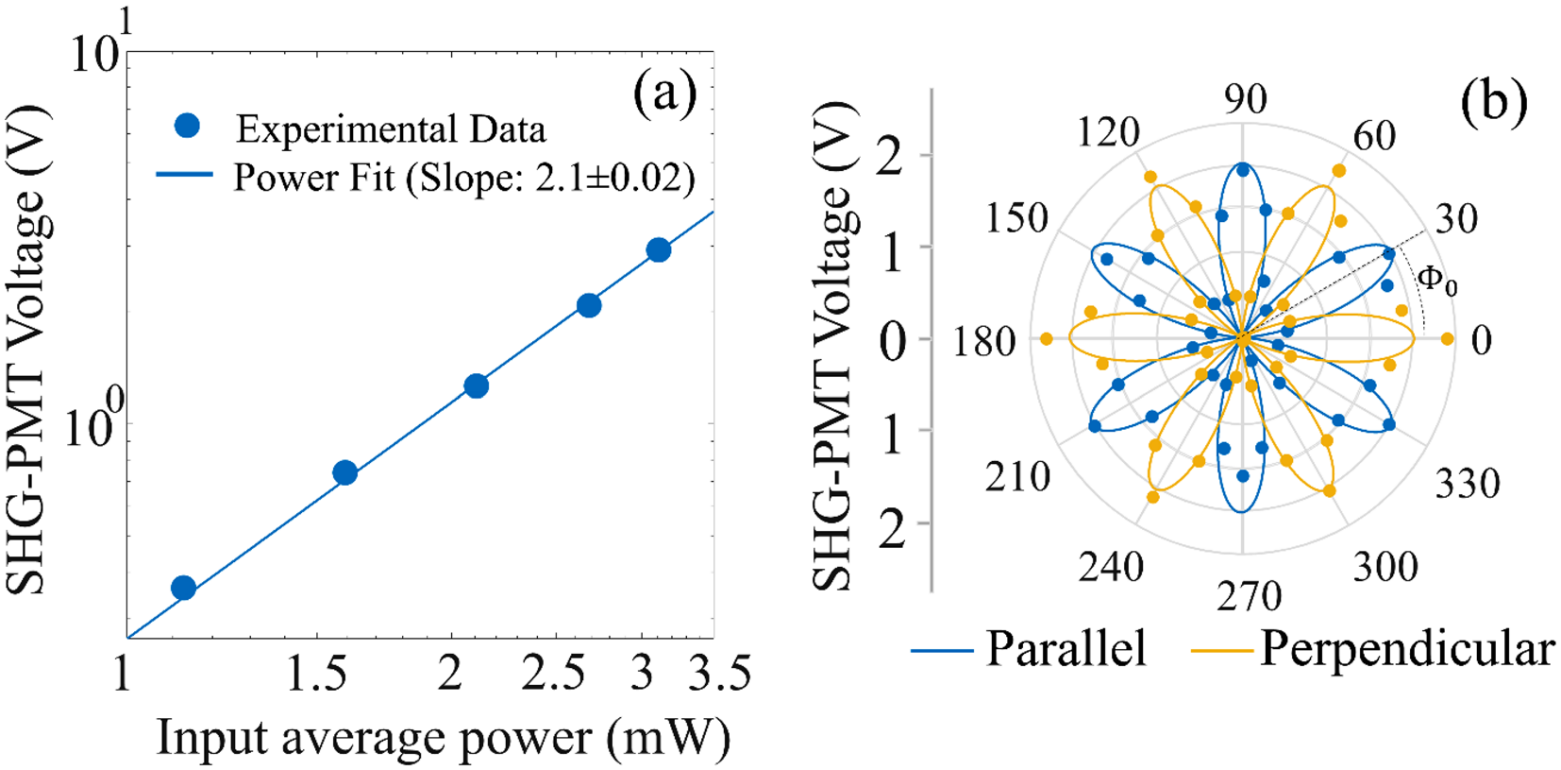
(**a**) Power dependence of SHG measurement. Filled circles correspond to experimental data and solid line corresponds to the linear fit of the log–log plot. The extracted slope is shown in the legend. (**b**) Polarization-dependent SHG measurement as a function of incident polarization direction. ${\phi }_{o}$ refers to the initial offset between the armchair direction and the laboratory axes. Blue and orange filled circles correspond to experimental data for parallel and perpendicular polarization relative to the analyzer orientation. Solid curves correspond to theoretical fit to the experimental data points. (Created in MATLAB v9.6, R2019a, https://in.mathworks.com/).

A summary of images obtained from four different multilayer 2H SnSe_2_ samples (labeled sample 1, 2, 3 and 4) is shown in Fig. 2. The sample preparation technique is described in the “Methods” section. Optical images obtained using reflectance microscopy are shown in Fig. 2a–d with the observed color contrast corresponding to the thickness dependence of the reflected light. AFM images of the same samples are shown in Fig. 2e–h. From AFM measurements, the thickness of the samples are in the range of 5–40 nm. We could not obtain monolayer or few layer SnSe_2_ samples in this study. SHG microscopy images of the samples are shown in Fig. 2i–l. The nonlinear optical microscopy system used for acquiring the SHG images is described in “Methods” section. The SHG images shown are acquired with fundamental excitation at 1040 nm, linearly polarized and parallel to the armchair axis of the SnSe_2_ flake (the armchair axis is indicated by the double-sided arrow in the SHG images). Qualitative inspection of the SHG images shows that the SHG signal is found to be highest for the thinner regions. We also observe rapid changes in the SHG signal for small changes in the flake thickness. This is clearly seen from the line scans included as insets in the SHG and AFM images. Such rapid variation in the SHG signal strength points to strong layer number dependence of the nonlinear signal. Figure 3a shows the quadratic functional form of SHG signal as a function of fundamental excitation power for sample 1. The slope of the log–log power dependent SHG signal plot is obtained as 2.1 ± 0.02, confirming the second-order nature of the generated nonlinear optical signal. Figure 3b shows the polarization dependent SHG from sample 1 obtained by rotating the sample from 0° to 360° for linearly polarized fundamental excitation with parallel and perpendicular analyzer orientations relative to the incident light polarization. The six-fold symmetry of the SHG signal in the polar plot can be attributed to the underlying hexagonal lattice system of 2H SnSe_2_ with D_3h_ symmetry^3^.

**Figure 4:**
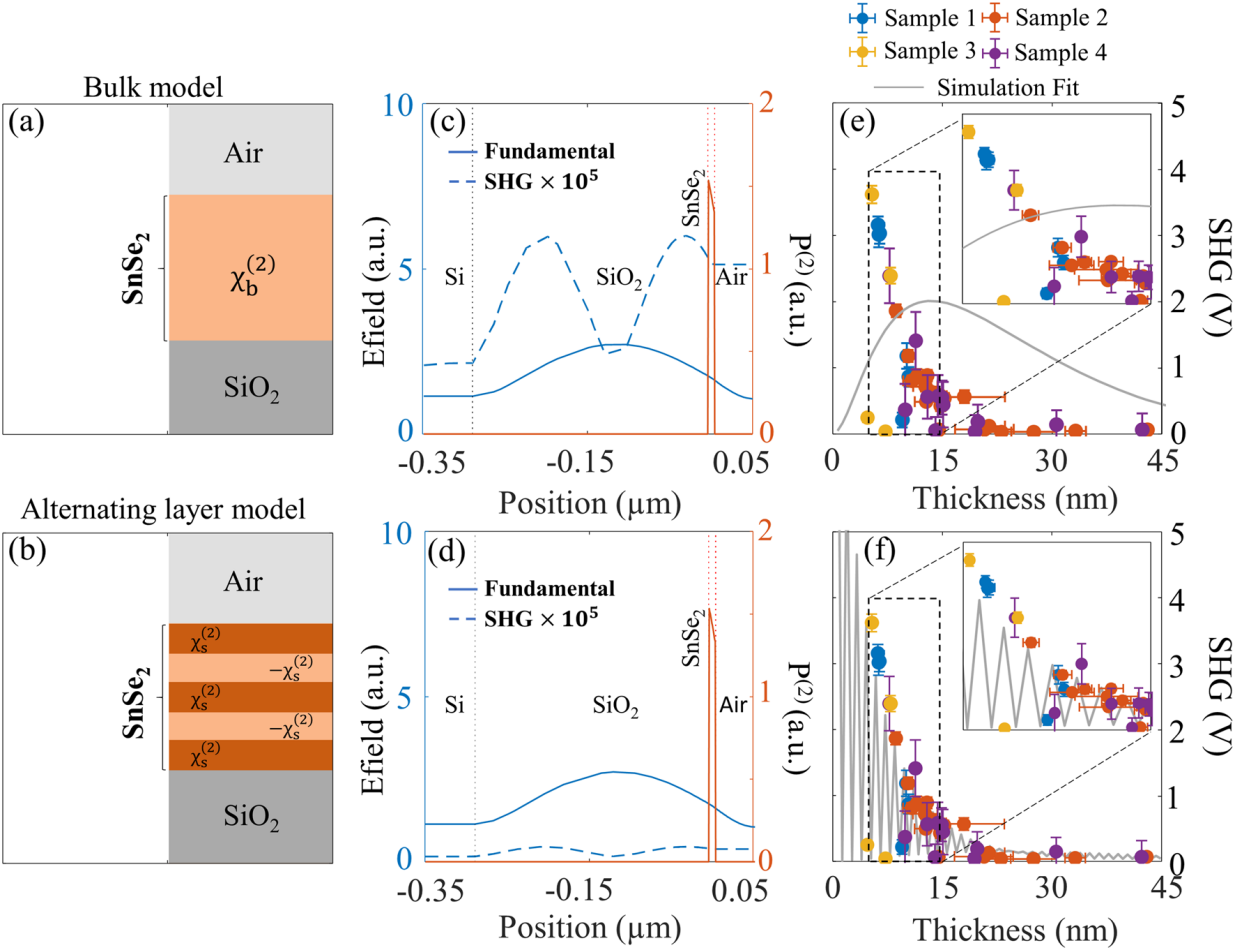
Comparison of thickness-dependent SHG experiments with nonlinear wave propagation simulations for multilayer SnSe_2_. Schematic cross-section view of the: (**a**) bulk nonlinearity model, and (**b**) alternating nonlinear layer model. (**c**, **d**) Simulation results showing the fundamental field profiles (solid blue curves), SHG field profiles (dashed blue curves) and nonlinear polarization (solid red curves). (**e**, **f**) Comparison of the experimental (solid colored circles) and simulated (solid blue curves) SHG signals for the two different nonlinearity models considered here. A zoomed-in view of this plot is shown as an inset. (Created in MATLAB v9.6, R2019a, https://in.mathworks.com/).

To understand the observed thickness dependence of SHG for 2H polytype of SnSe_2_ we utilize a nonlinear wave propagation model across the layered medium. The nonlinear wave propagation model implemented in COMSOL Multiphysics software^29^ is described in the “Methods” section. SnSe_2_ possess a large refractive index (close to 3)^12,30^ at the wavelengths of interest in this work, resulting in the SHG process being strongly influenced by Fabry–Perot effects in addition to the expected dependence on phase mismatch and optical absorption^19,31,32^. The nonlinear wave-propagation model inherently considers these effects when solving for the field profiles and detected nonlinear signal. Figure 4a, b show the schematic cross-sectional views of the layered material above a silicon dioxide layer on a silicon substrate for the two different nonlinear optical susceptibility models considered here. Figure 4a shows the bulk nonlinear optical susceptibility model with the SnSe_2_ layer having uniform second-order nonlinear optical susceptibility of ${\chi }_{b}^{\left(2\right)}$ across its thickness. Figure 4b shows the alternating layer model consisting of each monolayer of SnSe_2_ with alternating signs for the nonlinear optical susceptibility. This closely models the D_3h_ crystal symmetry with alternating signs of nonlinear polarization from adjacent monolayers^3,33^. The sheet nonlinear optical susceptibility, ${\chi }_{s}^{\left(2\right)}$ for the monolayer is given as: $\frac{{\chi }_{b}^{\left(2\right)}}{{L}_{eff}}$, with monolayer thickness *L*_*eff*_ = 0.65 nm. Figure 4c, d shows the simulated field profiles and nonlinear polarization obtained by solving for the nonlinear wave propagation equation for the two different models for a fixed SnSe_2_ layer thickness of ~ 8.45 nm (13-layers). Both the fundamental and SHG fields are shown with each region demarcated by vertical dotted lines and appropriate labels. The fundamental fields, as depicted by the solid blue line remain identical for both the models as this depends only on the linear optical properties of the 2D material. The solid orange lines represent the absolute value of the SHG nonlinear polarization. The SHG field shown by the dashed blue curve is however found to be lower for the alternating layer model (shown in Fig. 4d) when compared to the bulk model (shown in Fig. 4c). This is expected due to the approximate cancellation of the second-harmonic sheet dipoles resulting in SHG signal from effectively a single layer in the case of odd number of SnSe_2_ layers. In contrast, for the bulk model the SHG field is found to be significantly higher across the entire SnSe_2_ layer. Figure 4e, f show a comparison of the simulated SHG obtained at the detector plane with the experimentally obtained thickness-dependent SHG signal. The experimentally obtained thickness dependent SHG data from all the four samples shown in Fig. 2 are combined in this plot. The rapid oscillations in SHG signal combined with the gradual decrease with thickness are found to be in very good agreement with the alternating sign nonlinear susceptibility model (shown in Fig. 4f). In contrast, the simulated SHG signal for the bulk model peaks for a film thickness of ~ 10–15 nm and decreases slowly under the combined influence of phase mismatch and optical absorption (shown in Fig. 4e). This thickness-dependent SHG for the bulk model does not agree with the experimental data. The observed rapid variation in SHG signal for small changes in flake thickness, the good agreement with the alternating sign nonlinear susceptibility model, the measured six-fold polarization dependence of the SHG signal and observed spectral position of E_g_ Raman mode provides strong evidence for the observation of SHG from the 2H polytype of SnSe_2_ in this study with D_3h_ (D_3d_) symmetry for odd (even) numbered layer.

We also consider the possibility of interfacial break in symmetry contributing to the observed SHG signal from SnSe_2_, as observed in other centrosymmetric materials^34,35^. For pristine, dangling bond free surfaces achievable with mechanical exfoliated Van der Waal layered materials, the interfacial break in symmetry is in general not observed^36^. Furthermore, the interfacial model cannot explain the observed rapid oscillations in the SHG signal for small thickness variations as the SHG signal would exist irrespective of the layer number. SHG emission is also observed from distorted 1-T′ layered materials, such as Rhenium disulfide (ReS_2_) which otherwise exhibit centrosymmetry in the absence of lattice distortion^37^. There is however anisotropic polarization dependent SHG observed in the SHG emission due to lattice distortions. Given the symmetric sixfold polar plots obtained for SnSe_2_ as shown in Fig. 3b, lattice distortion can clearly be ruled out as the cause of SHG signal.

### Extracting second order nonlinear optical susceptibility of 2H SnSe_2_

**Figure 5:**
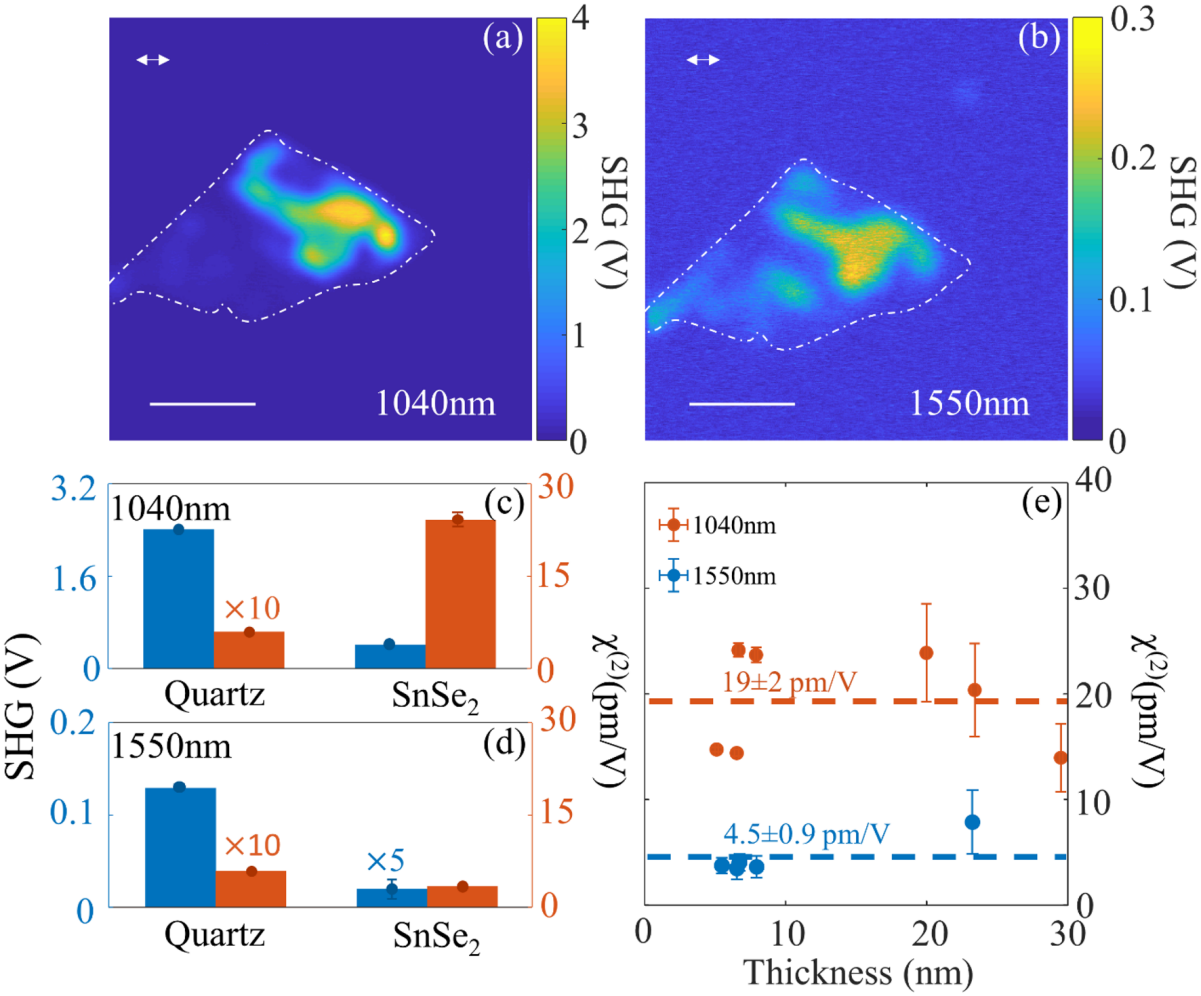
Wavelength dependent SHG from multilayer SnSe_2_. SHG images acquired at (**a**) 1040 nm, and (**b**) 1550 nm excitation wavelengths. The scale bar corresponds to 5 μm. Bar-charts comparing SHG signal (blue) and extracted nonlinear optical susceptibility (orange) for Quartz and SnSe2 for (**c**) 1040 nm, and (**d**) 1550 nm excitation wavelengths. (**e**) Thickness dependent nonlinear optical susceptibility for 1040 nm (orange) and 1550 nm (blue) excitation wavelength. The dashed lines indicate the average value. (Created in MATLAB v9.6, R2019a, https://in.mathworks.com/).

Next, the wavelength dependence of the SHG signal and calculating the second order nonlinear susceptibility from SnSe_2_ are investigated. Figure 5a, b shows the SHG images acquired from sample 3 at 1040 nm and 1550 nm fundamental excitation wavelengths. The SHG signal obtained for 1040 nm excitation is found to be ~ 175.5 times higher than that for 1550 nm excitation, when scaled to the same intensity levels and detector response. This enhanced SHG signal strength is attributed to the stronger nonlinear optical response for 1040 nm fundamental excitation wavelength. We also extract the second-order nonlinear susceptibility, χ^(2)^ at these two wavelengths by comparing the SHG signal from SnSe_2_ with a standard Z-cut quartz window. The procedure followed to extract the nonlinear optical susceptibility is described in the “Methods” section. Figure 5c, d shows a comparison of the SHG signals and extracted χ^(2)^ for the two excitation wavelengths and fixed SnSe_2_ layer thickness of ~ 7.1 nm (11-layers). The SHG signal from quartz is found to be ~ 5.8 and ~ 32 times higher than that of SnSe_2_ for 1040 nm and 1550 nm excitation respectively. Nonetheless, the χ^(2)^ values are found to be 32 and 7.5 times higher for SnSe_2_ when compared to quartz. In comparison to quartz, the nonlinear optical susceptibility for SnSe_2_ is higher even though the SHG signal strength is weaker due to the reduced interaction length over which SHG is generated for SnSe_2_. We also extend the χ^(2)^ coefficient calculation for varying SnSe_2_ thickness for the two excitation wavelengths in Fig. 5e to test the robustness of the χ^(2)^ calculation procedure. The observed variations in the χ^(2)^ value are mainly attributed to the error in AFM measurements leading to inaccuracies in the SnSe_2_ layer thickness considered in the calculation. The average χ^(2)^ values extracted for SnSe_2_ are 19 ± 2 pm/V and 4.5 ± 0.9 pm/V for 1040 nm and 1550 nm excitations respectively. The SHG signal strength and second-order nonlinear optical susceptibility for 1040 nm excitation are higher than that at 1550 nm excitation. This is attributed to the near band-edge excitation at 1040 nm, which is expected to enhance the nonlinear optical response from SnSe_2_, in similar lines to previous observations in direct and in-direct bandgap semiconductors^10,11^. The use of a fixed center wavelength fiber laser at 1040 nm in this study prevented the tuning of the excitation wavelength around the indirect band-edge of SnSe_2_ to obtain the nonlinear optical susceptibility spectrum.

### SHG from monolayer-MoS_2_/ multilayer-SnSe_2_ heterostructure

**Figure 6:**
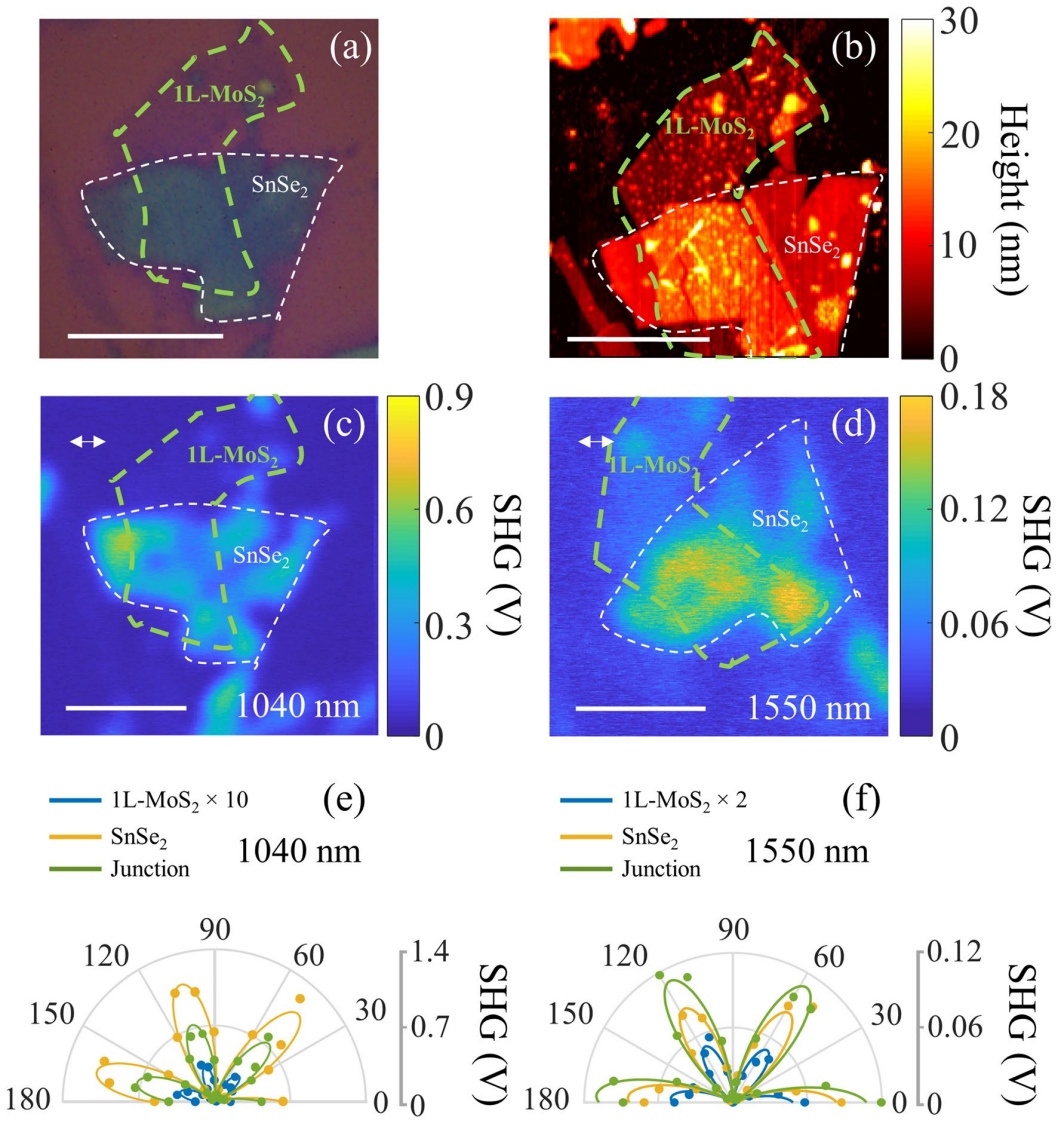
SHG from monolayer (1L) MoS_2_/multilayer SnSe_2_ heterostructure. (**a**) Optical brightfield image of the heterostructure with green and white dashed boundaries delineate the boundary of MoS_2_ and SnSe_2_ respectively. (**b**) AFM image of the heterostructure. SHG images acquired for (**c**) 1040 nm, and (**d**) 1550 nm excitation wavelengths. The scale bar in the images corresponds to 5 μm. Polarization dependent SHG polar plots shown for (**e**) 1040 nm and (**f**) 1550 nm excitation wavelengths. The polar plots corresponding to MoS_2_ region (blue curve), SnSe_2_ region (orange curve) and overlap junction region (green curves) are shown. The experimental data and six-fold fit are shown by filled circles and solid curves respectively. (Created in MATLAB v9.6, R2019a, https://in.mathworks.com/).

The nonlinear optical susceptibility values extracted here are comparable to previously reported values from other popular TMDC based layered materials^1,3^. However, large variations are observed in the nonlinear optical signal strength and susceptibility values reported previously^1^. Here, we perform SHG microscopy and polarization studies on artificially stacked heterostructures of monolayer MoS_2_ transferred on multilayer SnSe_2_, with the objective of comparing the relative SHG signal strength of SnSe_2_ with MoS_2_ in one sample and also understand SHG interference effects^38^ in the overlap region. The method followed for preparing the heterostructure is discussed in the “Methods” section. Figure 6a, b shows the optical and AFM images of the MoS_2_/SnSe_2_ heterostructures studied here. The green and white boundaries in Fig. 6a delineate the MoS_2_ and SnSe_2_ layers respectively. Figure 6c, d show the SHG images of the heterostructure acquired for 1040 nm and 1550 nm excitation wavelength. The SHG signal from SnSe_2_ is found to be ~ 34 and 3.4 times higher than MoS_2_ for 1040 nm and 1550 nm excitation wavelengths respectively. This is observed despite considering monolayer MoS_2_, which is expected to result in the highest SHG signal for layered MoS_2_^3^. The large SHG signal observed for multilayer SnSe_2_ when compared to MoS_2_ for 1040 nm excitation is attributed to the near-indirect band-edge excitation of SnSe_2_, with both fundamental (1040 nm/1.19 eV) and second harmonic wavelength (520 nm/2.38 eV) lies much far away from the excitonic resonance features in the case of MoS_2_ monolayer with a direct band gap at 1.9 eV. We also perform polarization dependent SHG studies on the MoS_2_/SnSe_2_ heterostructures to obtain the orientation dependence of SHG signal from individual layers. Figure 6e, f shows polar plots corresponding to the polarization dependence of SHG signal with the sample rotated from 0° to 180° with the incident light linearly polarized and aligned parallel to the analyzer direction. The polar plots show the SHG signal obtained from SnSe_2_, MoS_2_, and the overlap regions. The polar plots show the expected six-fold symmetric pattern when considering the full 0°–360° polarization angle. This agrees with the expected SHG polarization dependence for material with D_3h_ symmetry, such as SnSe_2_ and MoS_2_. The SHG images at 1040 nm and 1550 nm excitation wavelength are rotated by 60° and are equivalent in terms of the armchair orientation relative to the incident polarization direction due to the six-fold symmetry. The arm-chair axes of MoS_2_ and SnSe_2_ layers are found to be aligned very close to each other during the layer transfer, as evident from the overlap of the six-lobe polar plots for the individual layers. The SHG signal from the overlap junction region is determined by the interference of the SHG generated from the individual layers and the relative phase between the individual second-order nonlinear polarization. The SHG signal from MoS_2_ being weaker than SnSe_2_ at 1040 nm excitation results in weak interference between the SHG generated between the two layers. The overlap junction region is found to show reduced SHG signal for 1040 nm excitation due to the absorption of the generated SHG at 520 nm by the MoS_2_ overlayer. In contrast, the overlap junction region shows enhanced SHG signal for 1550 nm excitation, which is attributed to stronger interference due to comparable SHG signals for the individual layers and negligible reabsorption in the MoS_2_ layer. Previous reports have shown enhanced photoluminescence^18^ and Raman scattering^39^ from the overlap region of MoS_2_/SnSe_2_ heterostructures. This is attributed to a resonant energy transfer mechanism between the SnSe_2_ layer and the MoS_2_ layer. In the context of parametric nonlinear optical processes such as SHG, we do not expect to observe enhancement in the overlap region due to resonant energy transfer processes. This is due to the instantaneous nature of the parametric nonlinear process such as SHG, resulting in negligible enhancement due to the more slower energy transfer mechanisms.

## Conclusions

We report, for the first time to the best of our knowledge, SHG from multilayer 2H polytype of SnSe_2_ and observe strong second-order nonlinear optical response for fundamental excitation in the vicinity of the indirect band-edge. Comparison between the SHG and Raman spectra for two different polytypes of SnSe_2_ shows strong(negligible) SHG combined with characteristic E_g_ Raman modes at 109 cm^−1^(119 cm^−1^) for the 2H(1T) polytypes. The Raman peak position difference between the A_1g_–E_g_ modes also shows clear thickness dependence for the 1T form, which is absent for the 2H form. We observe rapid variations in the SHG signal for small changes in flake thickness and characteristic six-fold symmetric polarization dependence of SHG. The thickness dependence of SHG signal is found to be in good agreement with a nonlinear wave propagation model considering multiple monolayers of alternating signs for the nonlinear susceptibility. The nonlinear optical susceptibility of SnSe_2_ is found to be more than four times higher for 1040 nm when compared to 1550 nm excitation. This is attributed to the strong nonlinear optical response close to the indirect band-edge of SnSe_2_. We also acquire SHG images from monolayer MoS_2_/ multilayer SnSe_2_ sample to unambiguously compare the relative SHG signal strengths and understand the interference effect in the overlap region. We find that the SHG signal and hence the interference signal in the overlap region is dominated by the SnSe_2_ layer for the wavelengths under consideration. The present work shows that emerging 2D materials such as SnSe_2_ exhibit strong nonlinear optical response close to its band-edge even in the absence of excitonic resonances with nonlinear optical properties comparable to other popular 2D materials. The near-band edge enhancement of nonlinear optical response from such emerging 2D materials can potentially open the spectral range over which 2D materials can find applications as ultrathin nonlinear photonic devices. There is interest in realizing active photonic functionalities using 2D materials away from strong resonance features^40,41^. Having quantitative knowledge of the nonlinear optical properties close to and far away from band-edge or resonances as presented here is essential in evaluating the usefulness of these materials.

## Methods

### Sample preparation and characterization

Multilayer SnSe_2_ (from 2D Semiconductor, USA and HQ graphene, Netherlands) samples are prepared using dry transfer method in which Scotch tape is used to exfoliate the flakes of SnSe_2_ onto the Polydimethylsiloxane (PDMS) substrate. Suitable flake with varying thickness is identified and transferred by stamping the PDMS onto silicon substrate with 285 nm silicon dioxide layer. MoS_2_/SnSe_2_ heterostructure samples are prepared by first identifying uniform thickness SnSe_2_ layer with thickness of ~ 8 nm. Monolayer MoS_2_ is then transferred on top of the SnSe_2_ layer, partly covering the SnSe_2_ region. The final sample consists of monolayer MoS_2_/ multilayer SnSe_2_ junction region along with separate regions of monolayer-MoS_2_ and SnSe_2_ only for SHG studies. Photoluminescence (PL) measurement performed on the MoS_2_ layer showed PL peak position at ~ 1.9 eV, confirming monolayer MoS_2_ (not shown here)^18^.

Optical reflectance microscopy of the transferred flakes is performed using an inverted microscope, Olympus IX73. AFM imaging is performed using Park AFM NX20 system in non-contact mode. Raman spectroscopy is performed using Renishaw Raman spectrometer. All measurements are taken over a scanning range of 100–500 cm^−1^ with 532 nm excitation focused using a 100 × objective lens. The incident optical power at the sample is kept as low as 80 μW to avoid any heating effects on the sample.

### Nonlinear microscopy system

For the SHG measurement on the 2D materials, we use a conventional nonlinear optical microscopy setup^19^. This system consists of a femtosecond pump laser source (Fidelity HP) at 1040 nm wavelength with pulse width of 140 fs, and repetition rate of 80 MHz. The power and the polarization of the input laser source are controlled using half-wave plate and polarizer. A portion of the pump laser source is used to pump an optical parametric oscillator (Levante-IR) tuned to generate signal at 1550 nm wavelength with pulse width of ~ 200 fs pulse width and repetition rate of 80 MHz. The SnSe_2_ sample is fixed at the center of the rotational mount placed over an XY motorized stage on an Olympus inverted microscope. The input laser source is focused on the sample using a 20x/0.75 NA Olympus objective. The average input power on the sample at 1040 nm and 1550 nm excitation wavelength are measured to be 3.6 mW and 8.6 mW respectively. The measured illumination at the back aperture of the objective is around 42% and 100% for 1040 nm and 1550 nm excitation wavelength leads to a spot size of 3.64 µm and 2.52 µm, corresponding to a peak input optical intensity of 3.08 GW/cm^2^ and 10.74 GW/cm^2^, respectively. The backward emitted SHG signal is collected using the same objective and separated from the excitation laser using dichroic beam-splitter. The SHG signal is further spectrally isolated using a series of bandpass (520 ± 20 nm for 1040 nm excitation and 785 ± 40 nm for 1550 nm excitation) and short pass filters (890 nm cut-off) and detected using a photomultiplier tube (PMT). The set of bandpass and short-pass filters ensured effective rejection of the fundamental excitation with ~ 150 dB extinction, thus ensuring detection of only the SHG signal. SHG images are obtained by scanning the incident laser beam using a pair of galvo-scanning mirrors and mapping the PMT signal. The use of a fixed fiber laser source at 1040 nm center wavelength prevented scanning the fundamental excitation wavelength. For SHG polarization studies, the 2D material sample mounted on a rotary mount is rotated from 0° to 360° in 5° steps. The parallel and perpendicular oriented SHG signals are detected by using an analyzer in front of the PMT. The parallel and perpendicular SHG components refer to the analyzer oriented parallel and perpendicular to the incident linear polarization directions respectively.

### Nonlinear wave propagation simulation

The evolution of the nonlinear optical signal in the SnSe_2_ samples and its radiation to the far-field is described using the inhomogeneous wave equation under the slowly varying amplitude approximation. This nonlinear wave propagation equation for the second harmonic field (${\mathrm{E}}_{2}$) can be expressed as^8^:

1 $${\nabla }^{2}{\overrightarrow{\mathrm{E}}}_{2}-\frac{\upepsilon \left(2\upomega \right)}{{\mathrm{c}}^{2}}\left(\frac{{\partial }^{2}{\overrightarrow{\mathrm{E}}}_{2}}{\partial {\mathrm{t}}^{2}}\right)=\frac{1}{{\upepsilon }_{0}{\mathrm{c}}^{2}}\left(\frac{{\partial }^{2}{{\overrightarrow{\mathrm{P}}}_{2}}^{(2)}}{\partial {\mathrm{t}}^{2}}\right)$$

Here, the second order nonlinear polarization at the SHG wavelength, ${{\mathrm{P}}_{2}}^{\left(2\right)}$ is given as: ${{\mathrm{P}}_{2\mathrm{i}}}^{\left(2\right)}={\upepsilon }_{\mathrm{o}}{\upchi }_{\mathrm{ijk}}^{(2)} {\mathrm{E}}_{1\mathrm{j}}{\mathrm{E}}_{1\mathrm{k}}$ is the source term for the SHG process which depends on the mixing of the fundamental input electric field components (${E}_{1j}{E}_{1k}$). The nonzero elements, ${\upchi }_{\mathrm{ijk}}^{(2)}$ for a crystal with D_3h_ symmetry are given as *ijk* $\equiv$ *yyy* = − *yxx* = − *xxy* = − *xyx*^3,7^ with *x*, *y*, *z* corresponding to the crystal coordinate system. The *x* and *y* axis refer to the zig–zag and arm-chair directions. The nonlinear wave propagation equation is solved using the wave optics module in COMSOL using the direct finite element method (FEM) solver in frequency domain^29^. The input fundamental excitation considered here is a focused Gaussian beam within the first solver. The spot radius and peak electric field are calculated and fed into the COMSOL model using the objective lens numerical aperture, incident average power and femtosecond laser specifications used in the experiments. The nonlinear polarization is defined only in the SnSe_2_ region as given by the equation above. This acts as the source for SHG emission for the second solver to solve for the second harmonic field. The propagating SHG field is collected at a monitor plane to calculate the overall SHG signal.

### SHG polarization studies

The six-fold polarization dependent SHG plot obtained for crystals with D_3h_ symmetry can be theoretically fitted with the SHG signal dependence with polarization angle given as: I_||_= I_0_ sin^2^(3θ + 3Φ_o_) and I_⊥_ = I_0_ cos^2^(3θ + 3Φ_o_) for parallel and perpendicular orientations respectively^3^. Here, θ refers to the rotation angle of the crystal axis with respect to the lab coordinates and Φ_o_ refers to the initial offset of the crystal axis with respect to the lab coordinates. For the nonlinear wave simulation, we simulated for I_||_ assuming θ = Φ_o_ = 0, considering the maximum SHG signal orientation along the arm-chair axis in order to explain the thickness dependence SHG and extract the nonlinear optical susceptibility. Thus, ${\chi }^{\left(2\right)}$ specified without subscripts refers to the ${\chi }_{yyy}^{(2)}$ component.

### Extracting the second order nonlinear optical susceptibility

To extract the second order nonlinear susceptibility of SnSe_2_, we first calculate the backward SHG emission from a Z-cut quartz sample with input gaussian beam focused within the thick quartz sample. Here, the non-resonant nonlinear susceptibility of quartz is taken as: ${\chi }_{quartz}^{\left(2\right)}$ = 0.6 pm/V and is considered approximately constant across the different excitation wavelengths considered^8^. The backward SHG emission from the SnSe_2_ layer is simulated using the alternating layer model as described in the “Results and Discussion” section for a range of values of nonlinear susceptibility (1–100 pm/V in a steps of 2 pm/V) for the same set of input source parameters as used for quartz SHG simulation. The backward simulated SHG ratio is obtained by taking a ratio of SHG emission from SnSe_2_ to quartz. The effective nonlinear susceptibility of SnSe_2_ is then extracted by comparing the simulated SHG ratio with the experimental SHG ratio. For all the simulation, the complex refractive index spectrum for SnSe_2_ is obtained from ref.^30^.
